# A case of Familial Mediterranean Fever presented with recurrent infection

**DOI:** 10.5339/qmj.2023.sqac.33

**Published:** 2023-11-26

**Authors:** Marzieh Tavakol, Matineh Nirouei

**Affiliations:** ^1^Alborz University of Medical Sciences Email: marziyeh.tavakol@gmail.com

## Abstract

Introduction: The periodic fever syndrome Familial Mediterranean Fever (FMF) is caused by mutations in MEFV, which promote inflammation and present with uncontrolled systemic and organ-specific inflammation that can resemble infectious conditions. It is diagnosed based on clinical criteria, including frequent symptoms such as abdominal and thoracic pain, family history, and response to treatment with colchicine, which is confirmed by genetic assessment. Herein, we present a case of FMF with a relatively uncommon presentation.

Case presentation: A 22-month-old female was referred to the allergy and clinical immunology clinic with eczematoid skin rashes. She was admitted to the hospital several times since the 2^nd^ day of life with different complaints like fever, seizure, and restlessness, and treated with the diagnosis of septicemia. Endoscopy and colonoscopy were done at the age of 6 months due to poor weight gain and bloody diarrhea, which revealed active crypt-destructive colitis with foci of erosion in favor of infectious or allergic colitis. The colon biopsy was negative for Cytomegalovirus (CMV). Regarding family history, she was the second child of the first cousins parents and had a healthy 12-year-old sister. There was a history of death for unknown reasons of her cousin, whose parents were related. The immunologic evaluation was done, which showed a relatively normal immunoglobulin level, antibody response, flow cytometry, and lymphocyte transformation test. Nonetheless, a genetic study was done, which showed a homozygote mutation in exon 10 of the MEFV gene in the patient and a heterozygote mutation in both her parents.

Conclusion: The periodic fever syndrome Familial Mediterranean Fever (FMF) is caused by mutations in MEFV, which promote inflammation and present with uncontrolled systemic and organ-specific inflammation that can resemble infectious conditions. The patient was diagnosed with FMF, and treatment was started using colchicine, which successfully controlled the patient’s symptoms and prevented the recurrence of fever and other inflammatory manifestations.

